# Automated Radiosynthesis of [^18^F]Fluoromannitol for Clinical Research on a Commercially Available Trasis AllinOne Radiosynthesizer

**DOI:** 10.21203/rs.3.rs-7124066/v1

**Published:** 2025-08-01

**Authors:** Amy L. Vavere, Allison J. Clay, Arijit Ghosh, Joana Marie Almazan, Melissa J. Brown, Spenser Simpson, Kiel D. Neumann

**Affiliations:** St Jude Children’s Research Hospital; St Jude Children’s Research Hospital; St Jude Children’s Research Hospital; St Jude Children’s Research Hospital; St Jude Children’s Research Hospital; St Jude Children’s Research Hospital; St Jude Children’s Research Hospital

**Keywords:** Radiosynthesis, Automation, Radiofluorination, Fluoromannitol, PET, Infection

## Abstract

**Background:**

Infections pose a significant risk to immunocompromised individuals, and accurate, efficient diagnosis remain challenging. Current imaging methods like MRI and FDG PET lack pathogen specificity which complicate diagnosis and lead to overuse of antibiotics. Recent data shows that [^18^F]fluoromannitol ([^18^F]FMtl) is sensitive and specific to infection in vivo by exploiting the pathogen-specific mannitol transporter. This work aims to establish a reliable, automated method for producing [^18^F]fluoromannitol to facilitate clinical research studies in human subjects.

**Results:**

This study optimized and automated the radiosynthesis of [^18^F]fluoromannitol ([^18^F]FMtl) on a Trasis AllinOne synthesizer. The 105-minute synthesis achieved an average yield of 11.0% (n=19) with >97% radiochemical purity, and the product remained stable for at least 8 hours. While yield was lower than the previously reported manual method, automation enabled reproducibility and sterility. Process improvements included optimizing evaporation steps and reaction temperature, which significantly increased fluorine incorporation and yield. The process was validated to meet USP <823> regulatory requirements including full QC testing on three consecutive batches.

**Conclusions:**

An automated method for the radiochemical synthesis of [^18^F]fluoromannitol was developed and optimized on a commercially available Trasis AllinOne radiosynthesizer. This method allows for the reliable production and global dissemination of [^18^F]FMtl for use in clinical research trials.

## Background

Infectious diseases have a major impact on the health and economies of countries worldwide, especially those with lower income. In the United States, the death rate from infection in cancer patients is nearly three times that of the general population (Zheng et al. 2021). In a study of pediatric cancer patients, infection accounted for half of treatment-related mortalities or death not due to progression of their disease (Loeffen et al. 2019). Diagnosis of infection relies on many variables, including clinical symptoms, invasive biopsies, laboratory tests, and pathology, all which can take several days. In the interim, patients are often given unnecessary cycles of antibiotics which the Center of Disease Control and Prevention (CDC) cites as the largest contributor of antibiotic resistance. The rising trend of antimicrobial resistance, compounded by a growing population of immuno-compromised individuals (HIV/AIDS, chemotherapy, organ transplant, diabetes), creates an enormous economic strain on the US healthcare system. Cost estimates range from $28-$45B annually, calling for an improvement in infectious disease management (Stone et al. 2009).

Immunocompromised individuals (e.g. cancer treatment, HIV/AIDS, organ transplant, diabetes) are at increased susceptibility to opportunistic infectious diseases. In fact, infection is one of the most common complications of cancer and cancer treatment (Seo et al. 2020). In addition, these individuals often have atypical symptoms which not only cause issues with diagnosis, but may delay care (McGrath et al. 2020). In a decade-long study in the Netherlands encompassing almost 1800 pediatric cancer patients, infection was responsible for roughly 53% of treatment-related mortality across all diagnoses (hematological, solid tumor, and brain tumor) (Loeffen et al. 2019). A Danish study of 3255 patients over twenty years concluded that infection was the leading cause of treatment-related deaths at 37% (Sorenson et al. 2024).

Infection can also cause extreme complications in patients with chronic conditions. Osteomyelitis is a significant complication for patients with sickle cell disease (Al Farii et al. 2020). These patients often also suffer from vaso-occlusive crises that can present in a similar fashion, and there is currently not a test or imaging method to clearly differentiate between the two (Berger et al. 2009; (Scruggs and Pateva et al. 2023). As a result, patients are often overprescribed antibiotics as a safeguard. Imaging can often be used to rule out other potential diagnoses but is not specific to identify infection.

Magnetic Resonance Imaging (MRI) is often employed in the evaluation of musculoskeletal concerns, and has a 100% negative predictive value in excluding infection in these tissues (Weaver et al. 2022). However, differentiating infection from the host response is not possible due to many similar radiographic features (Salaffi et al. 2021). Positron emission tomography (PET) offers an imaging modality that follows function with radiolabeled molecules of interest to map processes within the body. The most common clinical PET tracer for imaging infectious diseases is [^18^F]fluorodeoxyglucose (FDG), which allows interrogation of glucose metabolism over the entire body. FDG, while not specific for infection, has grown to be a valuable tool in the rapid detection of inflammation and infection, even gaining approval by CMS for reimbursement in these cases (Wahl et al. 2021). However, more specific tracers are essential, especially in confounding cases with differential diagnoses.

In recent years, a significant amount of preclinical research targeting bacterial-specific metabolic pathways has proven worthwhile to increase specificity of PET tracers for infection (Calabria et al. 2024; (Kahts et al. 2024; (Kleynhans et al. 2023). As a testament to the outstanding need for an imaging agent capable of specifically diagnosing bacterial infection in vivo, many recently developed radiopharmaceuticals seek to exploit various bacteria-specific signatures such as sugar/sugar alcohol metabolism (Gowrishankar et al. 2017; (Kang et al. 2020; (Li et al. 2018; (Ning et al. 2014; (Takemiya et al. 2019; (Weinstein et al. 2014), folic acid biosynthesis (Mutch et al. 2018; (Zhang et al. 2018), *D*-amino acid metabolism (Neumann et al. 2017; (Parker et al. 2020), and labeled antibiotics (Gowrishankar et al. 2017; (Sellmeyer et al. 2017; (van Oosten et al. 2013; (Zhang et al. 2016) in both normal and infected cohorts (Britton et al. 2002; (Ordonez et al. 2020; (Sellmeyer et al. 2017; (Tucker et al. 2018). Despite these scientific advances, there is a persistent and dire need for imaging agents that meet the challenges of clinical infectious diseases practice.

Recently, [^18^F]fluoromannitol was shown to be highly specific for detection bacterial infection in vivo exploiting mannitol transport (*mtl*A) not utilized by mammals. [^18^F]FMtl successfully distinguished inflammation from bacterial infection caused by *S. aureus*, *E. coli* and *A. baumannii* in multiple models of infection. [^18^F]FMtl also demonstrated the ability to monitor antibiotic efficacy of vancomycin treatment for *S. aureus* infections in vivo.

Based on these promising results, [^18^F]FMtl is being translated for clinical trials. As such, the goal of this work was to implement a routine, reproducible, and automated method for the production of [^18^F]fluoromannitol for clinical research studies. We sought to achieve this by using a Trasis AllinOne radiosynthesizer to result in sufficient activity yields to support clinical trials and a production time of less than two hours using a single-use manifold. We present here an optimized and validated radiosynthesis method in accordance with the guidelines of USP <823>, including quality control results from three consecutive successful syntheses as part of an investigational new drug (IND, #174021) application submission to the FDA.

## Methods

### General

All chemicals were purchased from Sigma-Aldrich (St. Louis, MO), unless otherwise noted. All aqueous solutions were prepared with ultrapure water (Milli-Q Integral Water Purification System, Millipore Corp.; 18.2 MΩ·cm resistivity) or sterile water for injection. The radiochemical precursor 4,6-O-Benzylidene-3-O-ethoxymethyl-2-O-trifluoromethanesulfonyl-1-O-methyl-β-D-glucopyranoside was obtained from ABX (Radeberg, Germany) and used as supplied.

**Figure 1:**
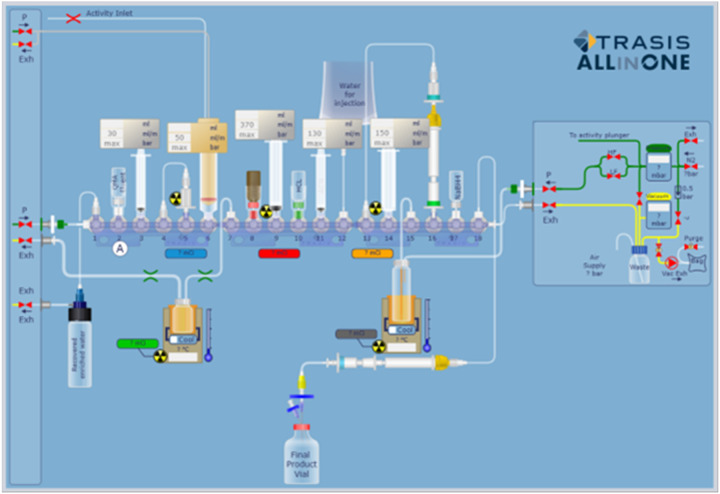
Custom manifold layout for Trasis AllinOne synthesis of [^18^F]fluoromannitol

The [^18^F]fluoromannitol ([^18^F]FMtl) synthesis method described was developed and optimized using an automated Trasis AllinOne radiosynthesizer and three, 6-port research cassettes custom assembled in series with additional tubing, syringes, and vials (see Figure 1). The assembly shown was used for a single production.

The final product line attached to the outlet of the manifold was cleaned and sterilized by rinsing with at least 10 mL sterile water, at least 10 mL sterile 70% ethanol, and then dried with nitrogen prior to each synthesis. All radioactive work was accomplished in a Comecer hot cell. The ^18^F-labeled materials were measured for activity using a Capintec CRC-15PET dose calibrator. Analytical HPLC was performed on a 1200 Series Agilent LC system using diode array detection, evaporative light scattering detection, and a Bioscan Flow-Count radionuclide detector (Eckert & Ziegler - Bioscan). Thin layer chromatography was assessed for activity using a Bioscan/Eckert & Ziegler AR2000 counter. Analysis for volatile organic impurities by gas chromatography was carried out using an Agilent model 8890 GC system. Both the LC and GC equipment were controlled by Agilent’s OpenLab CDS ChemStation software.

### Radiosynthesis of [^18^F]Fluoromannitol

**Figure 2:**
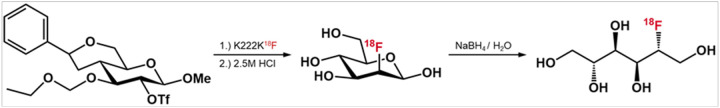
Radiosynthesis scheme of [^18^F]fluoromannitol

Briefly, [^18^F]FMtl was produced by fluorination of a benzylidene and ethoxymethyl-protected precursor followed by deprotection to produce [^18^]fluoromannose. This was purified, reduced and the final [^18^F]fluoromannitol was cartridge-purified following the work of Simpson et al. (Simpson et al. 2022) shown in Figure 2.

Aqueous [^18^F]fluoride, produced by the ^18^O(p,n)^18^F nuclear reaction in an IBA Cyclone^®^ 18/9 cyclotron from ^18^O-enriched water, was transferred from the target to a v-vial in a dose calibrator within the hot cell using argon flow (99.9999% high purity – Nexair). After measuring the activity value, the [^18^F]fluoride solution was passed through a QMA cartridge (Myja Scientific) to trap the [^18^F]fluoride and separate it from the [^18^O]water. The [^18^F]fluoride was then eluted from the QMA cartridge into the first reactor with a 1 mL solution of 9:1 acetonitrile-water containing 1 mg of potassium carbonate and 6 mg of Kryptofix (K222). The solvents were removed under nitrogen flow with reduced pressure and heating at 90°C for 4 minutes. A second azeotropic evaporation was performed by addition of 1 mL of acetonitrile followed by solvent removal under nitrogen flow with reduced pressure and heating at 90°C for 6 minutes.

Anhydrous acetonitrile (1 mL) containing 3 ± 0.3 mg of 4,6-O-Benzylidene-3-O-ethoxymethyl-2-O-trifluoromethanesulfonyl-1-O-methyl-β-D-glucopyranoside was added to the reactor vessel containing the dried [^18^F]KF. The vial was sealed and heated to 120°C for 20 min. After heating, the solvent was removed under reduced pressure with nitrogen flow at 120°C for 5 minutes. After the solvent was removed, 1 mL of 2.5 M hydrochloric acid was added to the reactor vial for deprotection, and the solution was heated to 150°C for 10 minutes to form the [^18^F]fluoromannose intermediate. The reactor vial was removed from heating and cooled for 1 minute.

Water was added to the reactor (2 mL), and the aqueous intermediate solution was passed through a cartridge purification system consisting of an Accell Plus CM plus short cartridge, followed by 3 g of AG^®^ 11 A8 resin [custom packed in a Phenomenex Strata C18-U (55 μm, 70 Å) cartridge], and a Sep-Pak Alumina N plus long cartridge all connected in series (previously conditioned with 20–30 mL of water per cartridge) and eluted to waste. An additional 6 mL of water was passed through the cartridge system and collected in a second reactor preloaded with 8 mg of sodium borohydride in 2 mL water. The reactor was sealed, and the aqueous solution was heated to 60°C for 30 minutes. The reactor vial was removed from the heating source and allowed to cool for 30 seconds.

The aqueous solution was transferred from the second reactor vial through a cartridge purification system consisting of a Chromabond Set V (previously conditioned with 30 mL of water), followed by a SCX cartridge (previously conditioned with 10 mL of water), and QMA plus cartridge (previously conditioned with 3 mL 1M sodium bicarbonate and 5 mL of water) all connected in a series and, finally, through a sterilizing filter (0.22 μm) into the sterile final product vial assembly. Lastly, 2 mL of water was passed through the cartridge series and collected into the final product vial for a final volume of 10 mL.

### Quality Control Testing & Validation

Several quality control tests were performed on the [^18^F]FMtl product to assure the quality of the final dose in accordance with guidelines from USP <823> Radiopharmaceuticals for Positron Emission Tomography-Compounding. The final product was confirmed to be clear and colorless with no visual evidence of cloudiness or particulate matter per USP <631> Color and Achromicity.

Due to the nature of this product and synthesis intermediates, a single analytical method cannot be used to quantify potential impurities, as some of the compounds interact with a C18 HPLC column, and some require a specialized ion exchange column specifically designed for sugars. For analysis with a standard C18 HPLC column, we used an Agilent Zorbax Eclipse XDB-C18 (4.6 × 150 mm) with an isocratic solvent system of 70% acetonitrile and 30% water at 1.0 mL/min. This allowed elution of precursor, partially deprotected precursor ((+)-(4,6-O-Benzylidene)methyl-α-D-glucopyranoside), benzaldehyde, and benzyl alcohol. For analysis of sugars and sugar-like impurities, we employed an evaporative light scattering detector (ELSD) to visualize compounds that do not contain chromophores for standard UV detection. We were able to get a reproducible signal for our standards using a HILIC ion exchange column (Shodex Asahipak NH2P-50 4E; 250 × 4.6 mm) in 85% acetonitrile/15% water solvent at a flow rate of 1 mL/min. The ELSD evaporator temperature was 90°C and the nebulizer temperature was 50°C with a gas flow rate of 1.20 SLM.

To determine radiochemical identity, the HPLC ELSD peak of the fluoromannitol standard in the coinject solution was compared to the activity peak measured by the radioactive detector (see Supplementary Information for example). To determine radiochemical purity, the area of the radioactivity peak corresponding to [^18^F]Fluoromannitol in the final product formulation represented not less than 90% of the total activity measured across the chromatogram for all other detected activity peaks. Three injections of a standard solution were performed on the same day, and the variation in area between injections was observed as verification of proper functioning of the HPLC instrument (system suitability). All standard areas had a relative standard deviation of ±10%. The ELSD spectrum of the final product formulation was used to estimate potential impurities. Since water has a spectrum on ELSD under these conditions, a water standard was injected. The sum of all ELSD peaks from the water standard were subtracted from the sum of all ELSD peaks in the final formulation. Any remaining area was compared to the average area of the three fluorodeoxymannose standards performed for system suitability to calculate an estimated mass of impurities.

To quantify the volatile organic impurities/residual solvents in the final sample, a small aliquot of the [^18^F]FMtl (0.5 μL) was analyzed by gas chromatography using a DB-WAX column (J & W 122–7032, 30 m × 250 μm × 0.25 μm). The oven temperature was set to 40°C for 1 minute, then ramped to 70°C at a rate of 20°C/min and held for 1 minute, then ramped to 80°C. The total run time was 3 minutes, and the front inlet heater was set to 250°C. The split ratio was 15:1, hydrogen flow was 40 mL/min, and the air flow was 400 mL/min. The GC peak retention times and areas were compared to standards of methanol (0.03%; RT ~ 1.68 min), ethanol (0.1%; RT ~ 1.88 min) and acetonitrile (0.03%; RT ~ 2.24 min). The amount of each component was calculated based on the ratio of peak areas for the samples vs. the standard.

Residual Kryptofix (K222) was determined by color spot test on silica based on the method by Mock et al. (Mock et al. 1997). The FDA has proposed a maximum permissible level of 50 μg/mL of Kryptofix^®^ 222 in 2-[^18^F]FDG, therefore we believe this maximum permissible level is appropriate for the [^18^F]FMtl final product. A strip of plastic-backed silica TLC plate was prepared by soaking in an acidic iodoplatinate solution and dried. 2 μL each of final product, water, and 50 μg/mL K222 standard was spotted on the iodoplatinated test strip. Kryptofix^®^ 222 in the standard will stain the center of the spot a dark color with a blue ring while the water standard will remain light in the center. The product was compared to the standard to confirm that the color change is lighter than the standard limit of 50 μg/mL. In addition, the pH of the final formulation of [^18^F]FMtl was tested by spotting on a narrow range pH indicator strip and comparing to the color range chart provided by the manufacturer. The final drug product was tested for the presence of bacterial endotoxins utilizing the Endosafe^®^-PTS^™^ unit (Charles River) to determine the endotoxin concentration in a sample. Sterility was tested using the direct inoculation method into trypticase soy broth and fluid thioglycolate media as recommended by the USP, and the samples were observed over 14 days.

The radionuclidic identity of the final product was determined by observing the radioactive half-life (t_½_) using a Capintec CRC-15PET dose calibrator with two measurements separated by an interval of 10 minutes. Radionuclidic purity was determined using a Canberra MCA System. Testing took place at least 8 days after production with an acquisition time of 12 hours, ensuring adequate spectral resolution for impurity identification. This approach follows standard industry practices, including USP <1821> Radioactivity, which requires sufficient decay time to detect potential long-lived impurities. The assessment was performed by the Cyclotron Facility at the Mallinckrodt Institute of Radiology, Washington University in St. Louis, using gamma spectrometry to compare the emission spectrum against known radionuclide decay schemes and calibration standards.

## Results

### Radiosynthesis of [^18^F]Fluoromannitol

**Figure 3:**
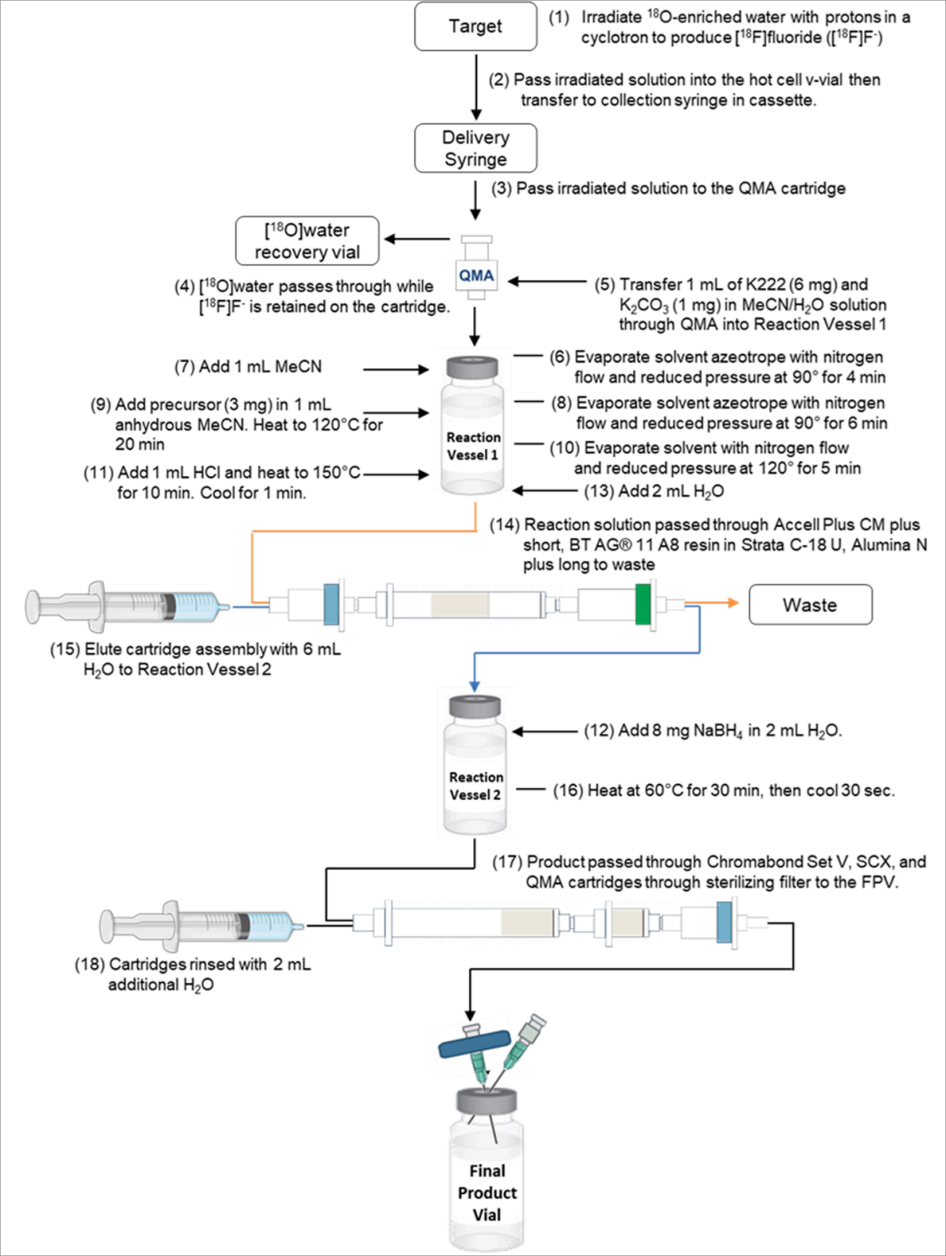
Schematic representation of the steps in the preparation of [^18^F]fluoromannitol

The goal of this study was to optimize and automate the synthesis of [^18^F]FMtl on a Trasis AllinOne radiosynthesizer, which, to our knowledge, has not been reported to-date. We based our synthesis script on the previously published method (Simpson et al. 2022) with some modifications and optimizations. Figure 3 shows a schematic representation of the steps in our radiosynthesis and purification of [^18^F]fluoromannitol.

The total synthesis time for [^18^F]FMtl was approximately 105 minutes and produced product with an EOS average non-decay corrected activity yield of 11.0 ± 2.12% (n = 19).

### Quality Control Testing & Validation

The synthesis routinely yielded product with > 97% radiochemical purity. Once all processes were optimized, three process validation syntheses were conducted to qualify the radiosynthesis for clinical research production and IND submission, and these results are shown in Table 1.

## Discussion

We based our synthesis protocol on previously published methods (Simpson et al. 2022) with some modifications (see Table 2.) to support automation. Final yield of the optimized synthesis of 11.0% is lower than reported manual synthesis yield of 23%, however the current reported method has the benefit of being fully automated and utilizing disposable, sterile cassettes.

During our optimization testing, we assessed whether the two additional azeotropic evaporations performed in the manual synthesis would result in higher initial fluorination on this system. In results not shown, we achieved a 25% increase in fluorine incorporation by adding a second azeotropic evaporation, but that benefit was lost with a third azeotropic evaporation. As a result, we added one additional evaporation. Preliminary optimization to reaction temperatures showed a doubling of overall activity yield by increasing the deprotection temperature from 140°C to 150°C (4.7 to 9.6%, n = 3–6). The reactor seal was compromised by raising the temperature to 160°C. Addition of a dose calibrator to measure exact F-18 starting activity was essential to allow an accurate determination of activity yield, which confirmed that we were previously underestimating. On a couple of occasions, we observed unexplainable, low trapping of F-18 on the QMA cartridge at the initial transfer to the radiosynthesizer resulting in a lower yield, however, final product concentration still allowed for patient dose preparation.

We opted not to include molar activity determination as previous work shows very low mass of fluoromannitol. In those studies, the measured mass concentration of fluoromannitol in the final product was 7.31 ± 0.25 μg/mL (n = 14). (Simpson et al. 2022) This meets the FDA microdose definition and is suitable for clinical research studies. *D*-mannitol is non-toxic to humans and the agent is FDA-approved for intravenous administration of concentrations as high as 25 g/100 mL of water, more than 34,000 times the mass reported in the final formulation of the radiopharmaceutical.

It should be noted that radiochemical purity and identity as well as chemical purity quality control testing of the final formulation of [^18^F]fluoromannitol are complicated by the fact that mannitol and fluoromannitol do not absorb UV light and are not retained on typical reversed-phase HPLC columns. Unfortunately, mannitol and fluoromannitol elute with the solvent front on these columns and therefore no separation of the product from [^18^F]F^−^ is possible to determine radiochemical purity. While other detector options are available, such as refractive index and evaporative light scattering detectors, they are not common in radiopharmaceutical labs. In addition, they often suffer from baseline drift, noisy background, and sensitivity to mobile phases making reproducibility challenging. In the context of quality control for release of PET tracers, these options offered many concerns. Initially, we considered a method whereby we confirmed identity via TLC analysis by comparing the R_f_ of our drug product (radioactivity detection) to a standard using TLC of copper ion-impregnated silica that allows retention of the fluoromannitol. Visualization of the standard was possible by treating the plate with a common stain (basic potassium permanganate) and heating to develop. For determination of radiochemical purity, we used the more sensitive method of HPLC using an ion chromatography column for sugars and sugar alcohols and used the primary radioactivity peak of the now confirmed fluoromannitol (by TLC). However, quantification of impurities was hindered by our original methods. We ultimately opted to introduce an evaporative light scattering detector (ELSD). During initial analyses, we did observe a significant unknown impurity. To remedy this, we added an additional SCX Sep-Pak to the final purification that successfully eliminated the impurity, but this lowered the pH below acceptable limits. The final QMA cartridge (pretreated with sodium bicarbonate) was added to raise the final product pH to levels required for injection.

While analysis by reversed phase chromatography with UV detection is not useful for determining the identity or purity of the final product, we thought it essential to assess for any impurities that could be visualized in this manner and not by the HILIC HPLC with ELSD. There are several purification cartridges in this synthesis, so we did not expect to observe impurities, and this was confirmed through several (n = 25) analyses. In all cases, virtually no evidence of impurity peaks was observed in the final product by elution on standard C18 column and reversed-phase system (see Supplementary Information). With confidence from these tests, we elected not include this analysis in routine quality control testing, however, will perform annually and did include in our validation syntheses.

As outlined in FDA’s guidance on solvents in pharmaceutical products, acetonitrile and methanol are Class 2 solvents and are limited to 0.041% for acetonitrile and 0.3% for methanol; acetonitrile is used in this synthesis and methanol is a possible side product. Ethanol is a class 3 solvent and is limited to 0.5%. Ethanol is used to sterilize the radiosynthesis system and is a possible side product. Organic solvents are used by commercial vendors in the preparation of purification substrates and cartridges used in this synthesis; while they remove > 99% of residual solvents, we observed ethanol, methanol, acetone and isopropanol in our product during synthesis optimization. Although in very small quantities, they were confirmed to be due specifically to residual solvents that eluted from these cartridges with the product. This was confirmed by GC analysis of water passed through each cartridge prior to preparation for synthesis compared to solvent standards. Our results showed < 0.01% ethanol and isopropanol on the Alumina N cartridge, < 0.01% methanol and isopropanol on the 11 A8 + C18-U cartridge, and no quantifiable solvent amounts from the Accell CM. The Chromabond cartridge contained trace amounts of acetone, < 0.01% ethanol, and 0.5–1% methanol in our analysis. Additional volume of water rinses in preparation of the purification cartridges eliminated virtually all of the solvents.

## Conclusions

In summary, an automated method for the radiochemical synthesis of [^18^F]fluoromannitol was developed and optimized on a commercially available Trasis AllinOne radiosynthesizer. This method allows for the reliable production of [^18^F]FMtl passing USP < 823 > quality control testing for use in clinical research trials in less than two hours.

## Supplementary Material

The online version contains supplementary material available at:

## Figures and Tables

**Table 1:** Validation production results

Test	Specification	Batch 1	Batch 2	Batch 3
**Total activity @ EOS**	N/A	**7733 MBq (209 mCi)**	**7696 MBq (208 mCi)**	**4399 MBq (118.9 mCi)**
**Concentration @ EOS**	N/A	**773 MBq/mL (20.9 mCi/mL)**	**769 MBq/mL (20.8 mCi/mL)**	**440 MBq/mL 11.9 mCi/mL**
**Visual Appearance**	Clear, colorless, and free of particulates	**Pass**	**Pass**	**Pass**
**pH**	4.5–7.5	**5.0**	**5.0**	**5.0**
**Radiochemical Identity & Purity**	≥ 90% [^18^F]FMtl	RCP: **98.23%**	RCP: **97.10%**	RCP: **100.0%**
	RT within 15% of std.	RT Std: **13.515 min**	RT Std: **13.213 min**	RT Std: **13.567 min**
		RT Rad: **13.389 min**	RT Rad: **13.061 min**	RT Rad: **13.420 min**
		Difference: **0.93%**	Difference: **1.15%**	Difference: **1.08%**
**Impurities (ELSD)**	≤ 100 μg impurities/dose	Impurities conc.: **23.71 μg/mL**	Impurities conc.: **21.07 μg/mL**	Impurities conc.: **20.96 μg/mL**
		Max dose volume: **4.2 mL**	Max dose volume: **4.8 mL**	Max dose volume: **4.8 mL**
**Radionuclidic Identity**	Observed t_½_ is 105 to 115 minutes	**111.66 min**	**111.61 min**	**108.7 min**
**Volatile Organic Impurities**	Methanol ≤ 0.3%	**< 0.001%**	**< 0.001%**	**0.004%**
	Ethanol ≤ 0.5%	**0.017%**	**0.019%**	**0.008%**
	Acetonitrile ≤ 0.041%	**< 0.001%**	**< 0.001%**	**< 0.001%**
**Bacterial Endotoxin (LAL)**	≤ 175 EU/dose	**< 2.50 EU/mL**	**< 2.50 EU/mL**	**< 2.50 EU/mL**
**Filter Integrity**	≥ 50 psi breaking pressure	**60.4 psi**	**60.0 psi**	**60.3 psi**
**Sterility**	no growth observed over 14 days	**Pass**	**Pass**	**Pass**
**Impurities (UV)**	≤ 10 μg/mL	**< 0.01 μg/mL**	**< 0.01 μg/mL**	**1.14 μg/mL**
**Radionuclidic Purity**	≥ 99.5% ^18^F	**100%**	**100%**	**100%**

In addition, the product was tested for stability over eight hours. Radiochemical and chemical purity analysis showed no discernable change over that time with all values remaining stable, as well as meeting all QC acceptance criteria at the conclusion of this time.

**Table 2:** Optimizations made to synthesis in comparison to previous work

Synthesis Step	Trasis AiO – Current Work	Manual Method (Simpson et al. 2022)
Additional azeotropic distillation	1–1 mL MeCN	2–1 mL MeCN
Acid deprotection	150°C	140°C
Intermediate transfer	2 mL added to HCl to transfer to cartridge purification No 0.45 cm filter	HCl solution passed directly through cartridge purification 0.45 filter post purification
Reduction	8 mg NaBH_4_ No stirring	5.5 mg NaBH_4_ Stirring
Final purification	Chromabond Set V cartridge + SCX cartridge + QMA cartridge	Chromabond Set V cartridge

## Data Availability

The data used and/or analyzed during the current study are available from the corresponding author on reasonable request.

## Abbreviations

CMS: Centers for Medicare & Medicaid Services
ELSD: Evaporative light scattering detector
EOS: End of synthesis
FDA: Food and Drug Administration
FDG: Fluorodeoxyglucose
FDS: Fluorodeoxysorbitol
FPV: Final product vial
FMTL: Fluoromannitol
H_2_O: Water
HCl: Hydrochloric acid
HILIC: Hydrophilic interaction liquid chromatography
HPLC: High performance liquid chromatography
IND: Investigational New Drug
K222: Kryptofix^®^ 2.2.2
K_2_CO_3_: Potassium carbonate
KF: Potassium fluoride
LAL: Limulus amebocyte lysate
M1PDH: Mannitol-1-phosphate dehydrogenase
MeCN: Acetonitrile
MRI: Magnetic resonance imaging
NaBH_4_: Sodium borohydride
PABA: *para*-aminobenzoic acid
PET: Positron emission tomography
RT: Retention time
TLC: Thin layer chromatography
USP: United States Pharmacopeia
UV: Ultra-violet
